# Goodenough-Harris Drawing a Man Test (GHDAMT) as a Substitute of Ages and Stages Questionnaires (ASQ2) for Evaluation of Cognition

**Published:** 2018

**Authors:** Nasrin BARAHENI, Seifollah HEIDARABADY, Shahrooz NEMATI, Morteza GHOJAZADEH

**Affiliations:** 1 Pediatrician, Tabriz Pediatric Health Research Center, Tabriz Child Developmental Center, Tabriz University of Medical Sciences, Tabriz, Iran; 2 Faculty of Education and Psychology, University of Medical Sciences, Tabriz, Iran.; 3 Associate Professor of Physiology School of Medical Sciences, Tabriz, Iran.

**Keywords:** Concurrent validity, The Goodenough-Harris drawing A Man Test, ASQ2, Cognition

## Abstract

**Objective:**

The main aim of the current research was evaluation of concurrent validity of the Goodenough–Harris Draw-A-Man Test (GHDAMT) with the problem-solving subscale of ASQ2 among children between 54-60 months old in Tabriz City, northwestern Iran.

**Materials & Methods:**

In this cross-sectional study, 136 males and 105 females were selected by simple random sampling from nursery schools in Tabriz City, northwestern Iran in 2014 and tested with GHDAMT and ASQ2 to compare the concurrent validity of these tests in evaluation of cognition. Data were analyzed using Pearson or Spearman correlation coefficients and SPSS.16.

**Results:**

The mean Intelligence Quotient (IQ) in girls was 128±18.18 and in boys 118±18.50, and the difference was statistically significant *P*<0.001. There was no statistically significant correlation between GHDAMT and ASQ2 .The statistical correlation was significant between IQ and mental age among children who had -2SD score in problem-solving subscale, but there was no statistical correlation between children who had -1SD score *P<*0.002. There was no statistically significant correlation between problem-solving subscale of ASQ2 and mental age and IQ.

**Conclusion:**

GHDAMT did not have acceptable validity and concurrent validity of the test was less than 0.3. So GHDAMT cannot be used as a substitute of ASQ questionnaire. However, the correlation of two tests in children with intellectual and developmental disability was significant. After doing more studies in further research, it is possible to use GHDAMT as a proper tool for cognition evaluation of these children.

## Introduction

Child development is an interesting and challenging topic for many different scientific disciplines, such as Pediatrics, Psychiatry, and Psychology, In recent years, trials have been conducted to achieve accurately and evidence-based information associated with developmental markers and normal and abnormal developmental processes (1). In this regard, parents are eager to get enough information to know whether or not their child is both developing and growing naturally. This is of particular importance in families with a history of developmental disorders and risk factors during pregnancy, such as preterm delivery, etc. (1).

The assessment of human abilities, including children, has always been one of the areas of interest to professionals in the fields of development and psychometrics. Various tests have been designed to study and evaluate cognitive, social and emotional abilities considering different theoretical perspectives (2). Assessment and diagnosing processes along with labeling the child in relation to his/her development process can have challenging outcomes for the family. If objective and accurate measurement tools are not used in the assessment processes, the delicate developmental points can be ignored on several occasions and planning and intervention processes for treatment can encounter with problem. Only less than half of the mild mental and growth failure and/or mild emotional-behavioral disorders in children can be diagnosed clinically and without the use of tools (3).

Different abilities are assessed in child development including gross and fine motor skills, personal-social skills, language and communication and problem-solving (cognition). Developmental disorders are identified through appropriate tests during the screening process. This is a process which can identify the child suspected to have developmental delays or failures at very early or hidden stages and need further assessment. Many tests and questionnaires aimed at diagnosing developmental delays in children throughout the world have been created and validated accurately and systematically (3). 

Among these tests, the Goodenough–Harris Draw-A-Man Test (GHDAMT) and the Ages and Stages Questionnaire (ASQ) are the developmental process tests used by experts to assess children's abilities (4). In the current study, GHDAMT was selected due to being easy, cost-effective and short. The goal was to assess outpatient children referred to the clinic or doctor's office when parents were concerned or the doctor suspected cognitive delays by using this simple, fast and reliable tool.

The formal use of drawing for psychological assessment began with Florence Goodenough, a child psychologist, in 1926. “Goodenough first became interested in drawing when she wanted to find a way to supplement the Stanford-Binet intelligence test with a nonverbal measure” (5). She believed that children draw what they know not what they see and that the nature and content of a child’s drawing are related to their mental development rather than other things (5). Many changes can be seen in children’s drawings of different ages and these changes are directly related to a child’s general intelligence. Her widespread studies on children’s drawings led to the first drawing intelligence test which named Goodenough Draw a Man test. Among the other psychologists interested in children’s development was Piaget who did many studies on human drawing (1956-1970) (6).

Over the years, the Goodenough Draw a Man test has been revised many times with added measures for assessing intelligence, but the origin of the test has remained unchanged. In 1949, some tried to introduce it as personality test by making changes on it (7). Harris later revised the test as GHDAMT (8). The test is one of the easiest, most practical and universal tests, the procedure is simple and requires little time and it is feasible in different locations and cost-effective. The test just needs the child’s cooperation and parents play no role. The purpose of the test is to assess child's development in cognition scale (4).

Since the image of a man is the same in all cultures and is not affected by educational experiences and family and cultural contexts and with respect to its less bias and lower costs, as it only requires paper and pencil, the test is still in use. In addition, the test is useful and effective for children with hearing damage and developmental and mental disability who cannot respond to instructions of other IQ tests (4).

The Ages and Stages Questionnaire (ASQ) is another developmental test to assess children's abilities designed by specialists at the University of Oregon in accordance with normal developmental processes. This test is easy, affordable and applicable in different locations (3). This questionnaire can be completed by parents at every level of education and except to the scoring and interpretation does not need a specialist. After scoring the answers and summing up, they are compared with pre-determined cut-off points and the child’s status is determined. The most important thing about the test is its continuity and the ability to repeat it at different ages and one of the important advantages of the questionnaire is parental involvement in screening their child’s development. The psychometric parameters of the ASQ 2 test were assessed in different studies, including studies in Australia and Denmark (9), and the results have been relatively good. The ability of the test to identify the developmental disorder is calculated at more than 96% (3).

One of the most important issues in the field of psychometrics is how to validate and use the ability measuring tests and their assessment in relation to individual’s abilities. Concurrent validity is one of the necessary assessments in the validation of the tests. Concurrent validity indicates that one test or measuring tool can be a proper substitute for another test or measuring tool (10). When reviewing the literature no evidence was found on the concurrent validity of the Goodenough - Harris test and ASQ, and, therefore, the present study was done to fill this research gap. 

## Materials & Methods

Multistage simple random sampling was used to select 241 children aged 54-60 months (136 boys and 105 girls) of whom 10 were mentally disabled from nursery schools of Tabriz, northwestern Iran as the study population in 2014.

The GHDAMT assesses child cognition; problem-solving subscale in the ASQ2 associated with cognition is also taken into consideration.

The researchers chose the age group 54-60 months because if the performance of a 54 months old child in ASQ2 is at – 2SD level (i.e., failed at the area of problem-solving subscale), the performance is almost equal to a 36 months old, which is the minimum age that children can be measured with the GHDAMT. A 36 months old child acquires the ability to draw a circle and sketch a man and gradually adds body parts and details (11). 

In the beginning, in respect to ethical concerns, the purpose of the study was briefly explained to all the parents participating in the study and the participants were promised that their information would be kept confidential in all articles and resources extracted from this study. There would not be any psychological and emotional consequences for them and their children. In addition, participants were free to withdraw at any time. Then the 54 months and 60 months ASQ 2 Questionnaire were given to the parents who had with respect, children at the age of 53-55 months and 59-61 months to complete at home. The Goodenough-Harris test was conducted at each nursery school after completing the questionnaire.

The scores of ASQ2 Questionnaire in the cognitive domain (problem-solving) were calculated and compared with cut-off point values standardized by the Iranian Ministry of Health and Medical Education and classified based on the scores. The passing score was between -1 SD and -2SD and failing score was less than -2SD.

The drawing test, considering the parts and graphic details, based on test instructions was scored between 0-1 (8) and mental age was estimated after summing up the tests based on Table 1, and child IQ was originally computed by taking the ratio of mental age to chronological (physical) age and multiplying by 100.

**Table 1:** Equivalents of mental age for acquired scores

MA	SCORE	MA	SCORE	MA	SCORE	MA	SCORE
13-0	40	9-9	27	6-6	14	3-3	1
13-3	41	10-0	28	6-9	15	3-6	2
13-6	42	10-3	29	7-0	16	3-9	3
13-9	43	10-6	30	7-3	17	4-0	4
14-0	44	10-9	31	7-6	18	4-3	5
14-3	45	11-0	32	7-9	19	4-6	6
14-6	46	11-3	33	8-0	20	4-9	7
14-9	47	11-6	34	8-3	21	5-0	8
15-0	48	11-9	35	8-6	22	5-3	9
15-3	49	12-0	36	8-9	23	5-6	10
15-6	50	12-3	37	9-0	24	5-9	11
15-9	51	12-6	38	9-3	25	6-0	12
		12-9	39	9-6	26	6-3	13

Children with physical disabilities who have difficulty using pencil were excluded from the study. The obtained data were studied using descriptive statistics methods (Frequency, percentage and mean ± SD). Pearson or Spearman correlation coefficients were used to calculating the correlation between the two tests. Then an independent *t*-test was used to compare the mean of the two groups, and SPSS16 (Chicago, IL, USA) was used for statistical analysis. The *P*-value of less than 0.05 was considered statistically significant.

## Results

Assessment of concurrent validity and computing of the two test's correlation by using of Pearson correlation coefficient (Table 2) revealed that statistical correlation between five different subscales of ASQ2 with mental age and IQ obtained from the Goodenough-Harris testis:

**Table 2:** Statistical correlation coefficients among 5 subscales of ASQ2 with IQ

Variable	Communication	Gross motor	Fine motor	Problem Solving	Personal-Social
IQ Pearson Correlation	.193	.208	.308	.037	.213
*P*-value	.003	.001	.000	.578	.001

There was a statistically poor significant correlation between the communication domain and IQ (r = 0.19, n= 241, *P* <0.003), the gross motor domain and IQ (r = 0.20, n= 241, *P*<0.001) and the personal-social domain and IQ (r = 0.21, n = 241, *P*<0.001). There was a statistically moderate significant correlation between the fine motor domain and IQ (r = 0.30, n = 241, *P*<0/001). No statistically significant correlation between problem-solving subscale and IQ among children with any mental disability was seen.

The Goodenough-Harris average IQ scores were 128.2 ± 18.18 in girls and 118.2± 18.50 in boys, which was statistically significant (*P* = 0.001). 

**Figure 1:**
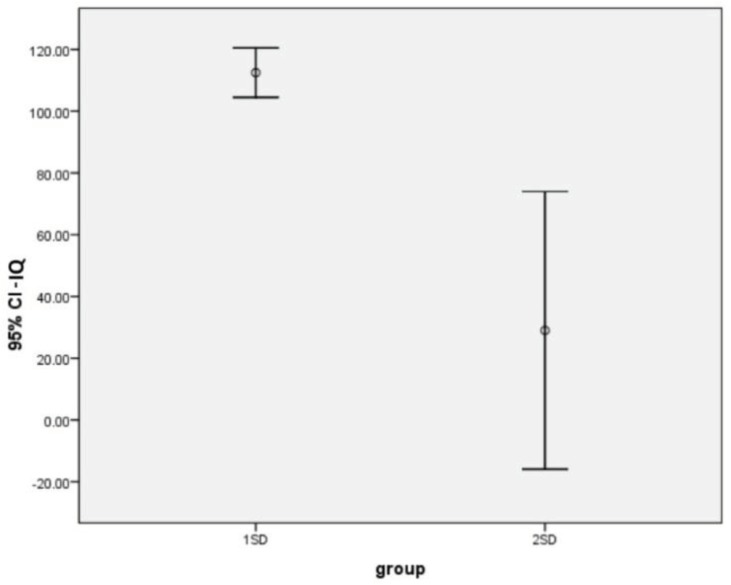
Error Bar graph. Comparing the IQ in two groups of children -1SD and -2SD

The results of the *t*-test (Figure 1) revealed that the average IQ scores of children who had -2SD score in solving problem subscale, and in children-1SD the difference was statistically significant (P=0.002).

There was no statistically significant correlation between ASQ2 and GHDAMT. A statistically significant correlation between IQ and mental age in the children who failed the problem-solving domain (less than -2SD) was seen *P*<0.002. However, the correlation was not significant in children whose ASQ2 score was higher than -2SD.

The reliability of GHDAMT was not acceptable and concurrent validity was less than 0.3. 

## Discussion

The aim of the current study was evaluation of the concurrent validity of two tests: the ASQ2 questionnaire and the GHDAMT. In order to compare these two tests, since GHDAMT assess the child’s cognition, the problem-solving subscale in ASQ2 questionnaire was selected. 

The results of current study suggest that the GHDAMT is not a proper substitute to ASQ2 in assessment of children’s cognition and revealed no statistically significant correlation between problem-solving subscale, mental age and IQ in children without mental disability. However, this correlation was significant in children with mental disability.

There are a lot of tests to assess child development that have been universally validated and used. However, they are often time consuming and require a professional to perform the tests and sometimes the high cost of the tests has led pediatricians to avoid using them and only relying on clinical diagnosis which in more than half of the cases, have led to misleading results and/or delay of early intervention (3). To solve this problem, more practical tests that are easy, short and cost-effective along with sufficient accuracy and validity are taken into consideration.

There are a few studies on the reliability and validity of the Draw a Man test, especially in recent years in Iran, but one was performed in Tabriz. In this study, in addition to GHDAMT, Raven's IQ Test was also done, which revealed acceptable reliability coefficients as well as good reliability and stability (12). The GHDAMT was done on children referred to an outpatient clinic and revealed that the test can be used as a useful tool in screening developmental disorders (13). However, this was not a valid test for assessing children’s IQ (4).

The ASQ2 test has been validated in global studies showing high reliability and accuracy (14). Parents re-did the test with 175 children in an interval of 2-3 wk, and this demonstrated the reliability of more than 94% of the results with a 0.1 SD. Re-testing was done on 112 children by an experienced person revealed more than 90% similar results (3).

To warrant concurrent validity, the ASQ2questionnaire was compared simultaneously to the following tests:

Revised Gesell and Amatruda Developmental and Neurological Examination

Bayley Scales of Infant Development

Stanford-Binet Intelligence Scale

McCarthy Scales of Children's Abilities

Battelle Developmental Inventory

There was almost 84% consistency between the results (3). The screening questionnaires completed by parents were as accurate as the ones done by pediatricians (15). The studies carried out on the ASQ2 in Iran verified its validity and reliability (16).

There are other researches questioning the validity of this type of questionnaires filled out by parents in public places (17). The ASQ2questionnaire was used on following up the program of preterm children up to 2 yr (19) and 5 yr (19) and was able to successfully identify the children with severe developmental delays. However, it was not efficient in identifying mild delays. The same test done in India on children of different ages, including high- and low-risk children has shown a sensitivity of 83.3% and specificity of 75.4% (20).

Concurrent validity of this test in following up of very premature children was also consistent with the Wechsler test (21). The efficiency of this easy and cost-effective test in assessment of developmental delay in high-risk children is reported (3).

**In conclusion,** GHDAMT cannot be a substitute for the ASQ2questionnaire due to the low statistical correlation coefficient. Although the statistical correlation coefficient was higher in children with mental disability but deciding, generalizing and judging in relation to the results is difficult due to the small sample size. The present study can be a starting point for further studies in evaluating IQ and cognition in children with mental disability.
